# Suitability and Potential Nutrient Contribution of Underutilized Foods in Community-Based Infant Foods in Northern Ghana

**DOI:** 10.3390/nu15112593

**Published:** 2023-06-01

**Authors:** Clement Kubreziga Kubuga, Cabral Bantiu, Jan Low

**Affiliations:** 1 Nutritional Sciences Department, University for Development Studies, Tamale P.O. Box TL1350, Ghana; bcabraldinho@yahoo.com; 2 International Potato Center, Nairobi P.O. Box 25171, Kenya; j.low@cgiar.org

**Keywords:** infant foods, underutilized crops, community-based, nutrient enrichment

## Abstract

In rural Ghana, infant feeding is largely home-based or community-based yet less is known about the kinds of community-based infant foods and the ability of families to create a range of recipes for baby feeding using context-specific ingredients particularly in northern Ghana which has a high prevalence of malnutrition. In this explorative study on mothers (15–49 years; *n* = 46), we investigated community-based infant foods’ food group composition, enrichment, nutrient contribution, and acceptability. The identified community-based infant foods were mainly made of either corn or millet porridges in northern Ghana and had three nutrients with % RNI ≥ 70. We developed 38 recipes of enriched community-based infant foods adding underutilized foods (orange-fleshed sweet potato, pawpaw, cowpea, moringa, groundnut, Bambara beans, and soya beans) to increase the number of nutrients from three to at least five and at most nine nutrients with % RNI ≥ 70 based on the recipe combinations. The enriched community-based infant food recipes provided adequate caloric amounts and modest improvements in micronutrient content for infants (6–12 months). All recipes tested were deemed appropriate and acceptable for infants by mothers. Moringa and pawpaw emerged as the lowest-cost ingredients to add among the underutilized foods. Future research is necessary to assess the effectiveness of the new recipes at promoting linear growth and improving micronutrient status during the complementary feeding period.

## 1. Introduction

Infant nutrition is a crucial component of a child’s development and health and the inadequate-quality diet of some young infants is a major public health concern [1]. For a child’s survival, growth, and development throughout the first two years of life, adequate intake of critical macronutrients and micronutrients is essential. The primary causes of so-called “hidden hunger”, driven by vitamin and mineral deficiencies in young children under two years of age, are non-exclusive breastfeeding and poor complementary feeding practices by caregivers [2]. Children between the ages of 6 and 23 months require supplemental nutrition in addition to breastfeeding. However, nutrient-rich complementary foods are not always affordable or available in low-income areas [3]. In many affluent societies, the intake of professionally produced infant food is very common and, in some cases, even outpaces that of home-cooked food. In rural Ghana though, infant feeding is largely home-based yet there is inadequate understanding of current and potentially enriched community-based infant foods using ingredients that can be grown or purchased in the local environment.

Infant foods created at home or at the community level typically contain ingredients that are tasty, fresh, and nourishing for kids [4]. Ghana, however, lacks national rules for the preparation of homemade infant food, despite the fact that they exist in several other nations [5,6,7]. While the lack of regulations may influence the ability to achieve optimal nutrition, the driving factors are poverty and lack of knowledge among caregivers [8]. Obviously, for rural households in Northern Ghana, if a food is being grown by the household, the potential for using it for infant feeding is higher than that for foods only available through market purchase. Moreover, the complementary feeding period is a crucial time in which to introduce infants to novel flavors and textures that will subsequently influence their preferences and acceptance of a wide range of well-known foods [9,10,11]. Good diet quality is best achieved by increasing dietary variety [12,13,14]. Additionally, there is proof that a lack of variety in a child’s diet during the first year of life may increase their chance of developing allergies and asthma later in life [13]. Unfortunately, data from Northern Ghana indicate that the average diet is monotonous and the supplemental foods given to young children are primarily cereal-based, no doubt helping to drive the high prevalence of malnutrition [15,16]. According to a recent micronutrient study, children in northern Ghana had a higher prevalence of stunting (25.3%), anemia (53.2%), and vitamin A deficiency (30.6%) compared to children in southern Ghana (16.6%, 32.3%, and 17.0%, respectively) [17]

Ghana’s infant and young child feeding is sub-optimal, particularly in the northern belt of Ghana [16]. Ghana did adopt the comprehensive Infant and Young Child Feeding strategy in 2007 [18], which includes actions to increase awareness through counseling and to provide support for adequate complementary feeding. However, there is little guidance on mothers’ or families’ self-efficacy in developing a variety of infant food recipes using context-specific foods.

In short, despite its significance, less is known about the kinds of community-based infant foods and about the ability of families to create a range of recipes for infant feeding using context-specific ingredients, particularly in northern Ghana, than is desirable [16,17]. For any CIF, whether currently existing or new, it would be important to know the nutrient contribution, the suitability and acceptance of that food by young infants and their caregivers. This study seeks to (1) document the common CIFs, (2) determine the acceptability and appropriateness of CIFs enriched with underutilized food crops, and (3) determine the nutrient contribution of underutilized food items (cowpea, orange-fleshed sweet potato (OFSP), pawpaw, soya beans, and moringa) to enriched community-based infant foods.

## 2. Materials and Methods

### 2.1. Study Design and Subjects

This explorative study used both qualitative and quantitative research methods when implementing community-based recipe trials. Key informant interviews (*n* = 9) and focus group discussions (*n* = 6) were used to obtain information on the kinds and composition of infant foods. This was followed by practical recipe trials to ascertain the acceptance, suitability and appropriateness of infant food recipes at the community level. The selection of underutilized crops was determined by identifying the major nutrient deficiencies reported by de Jager et al., 2014, namely vitamin A, vitamin C, calcium and vitamin B12, combined with assessing which crops and small livestock could meet these needs [19]. The recipe trials were conducted in two regions: the Northern Region (Kumbungu district—Kpulinyin and Dinyanpalgu communities), and the North East Region (East Manprusi district—Bongbini community) of Ghana from December 2021 through January 2022. This study used consumption data from an earlier study [20] that used the OPTIFOOD software (version 4.0.5) to model weekly community-based infant foods intake for children aged 6–12 months, with and without underutilized food items (cowpea, OFSP, pawpaw, soya beans, and moringa). Additionally, food price data of food items were taken from five local markets (cost/100 g), the Walewale, Tamale-Aboabu, Yendi, Damongo, and Saboba markets, during the harvest season (09/2022–10/2022) via a market survey, and mean price values were used in the model.

### 2.2. Recipe Development

Recipes were created with the intention of experimenting with ways to prepare readily available infant foods that are not typically given to infants and overcoming any resistance that may exist to their frequent use in Northern Ghana. Furthermore, these foods must to be suitable for infants, relatively cheap, and simple to make at home. Infant diets in rural Northern Ghana were primarily made from corn dough/flour or millet dough/flour, according to preliminary key informant interviews. In a few instances, women resorted to purchasing or making their own baby composite blend consisting of legumes, corn, and fish (which is relatively expensive). On this premise, a team from the University for Development Studies’ Nutritional Sciences Department created 38 enriched infant foods (recipes) using traditional CIFs as the base. The basic concept was to have at least two food groups per meal instead of the existing one food group per meal. The researchers were particularly interested in using underutilized food items (legumes, orange-fleshed sweet potato (OFSP), pawpaw, and moringa) to improve infant diets (porridges). Details for each enriched community based recipe are provided in the Supplementary Information (SP1).

### 2.3. Recipe Trials

Focus group discussions were held with participants (mothers: 15–49 years, *n* = 46; with children aged 6–12 months) in each community to inform researchers on existing CIFs. Six focus group discussions were held. Participants were asked the following questions: (1) Aside breastmilk, what kind of foods are given or prepared solely for children (aged 6–12 months) in your community? (2) Among the listed foods (if any), which one is commonly prepared for infants in your community? If more than two foods were reported, community members were asked to rank them in terms of frequency and the proportion of households that prepare them. After ranking the foods (if more than two), the most commonly prepared food was then selected for preparation and subsequent enrichment (using the developed recipes).

In each community, mothers (with children aged 6–24 months) were divided into groups of six each. Each group was asked and expected to provide the ingredients for commonly prepared infant foods in their respective communities. Mothers were tasked to prepare their homemade porridge for their children, the researchers prepared the same homemade porridge per the instructions of community members. Thereafter, food items (OFSP, pawpaw, cowpea, moringa, groundnut, Bambara beans, soya beans, millet dough, and corn dough) were displayed and mothers were asked if they had ever added any of the displayed food items to their homemade porridges. Participants were also asked if they could prepare any infant food with two or more of the displayed ingredients. If the response was affirmative, they were tasked to demonstrate by preparing the said porridge. The researchers led a cooking demonstration using the pre-developed recipes; other group members were provided the recipes and tasked to prepare them.

### 2.4. Community Based Testing of Foods

Paired preference testing was used to evaluate the developed recipes [21]. Each recipe was compared with the CIF in terms of appearance, aroma, sweetness, texture, and overall acceptability. Mothers’ opinions on acceptability and appropriateness of the prepared recipes for their children were sought. Acceptance of new recipes by children was tested according to consumption; i.e., if a child tasted a recipe 2–3 times consecutively without rejection, such recipes were considered to be accepted by the child.

### 2.5. Nutrient Adequacy of Community-Based Infant Foods

A nutrient-adequate diet (NA) is defined as the set of items available at each time and place that would stay within lower and upper bounds for dietary energy and all essential nutrients. To calculate the nutrient adequacy of community-based infant diets, we used a linear programing software (OPTIFOODs, version 4.0.9.0) to select food items to prepare different infant complementary foods to provide nutrient content that meets the needs of 50% of the healthy population of infants (aged 6–12 months), and the highest level of nutrients likely to prevent the risk of adverse health effects [22], while specifying that the macronutrient intakes are within the acceptable macronutrient distribution range, and meeting the energy content of exactly 654 kcal. Detailed methods have been published earlier [20]. Operationally, NA is a diet that meets the estimated energy requirement and relevant daily nutrient reference values of thirteen nutrients for a representative person in a reference population. The thirteen nutrients include total fat, total protein, calcium, vitamin C, thiamin, riboflavin, niacin, vitamin B6, folate, vitamin B12, vitamin A as RAE, iron and zinc. Models on infant intake were constructed using secondary data from an earlier study [20] conducted in the Northern Region.

### 2.6. Statistical Analysis

Data analyses were conducted using SAS (version 9.4, SAS Institute Inc., Cary, NC, USA) and OPTIFOOD software (version 4.0.9.0). The nutrient contribution of recipes was analyzed using OPTIFOOD and presented as % RNI. Sensory attributes were presented as frequencies and percentages.

#### Analysis in OPTIFOOD

With the help of the OPTIFOOD software, all analyses were performed using a three-module strategy to create weekly food-based recommendations (FBRs) for the target group; a community-based infant diet without underutilized food items, a community-based infant diet with one underutilized food item, and a community-based infant food with two underutilized food items were the three scenarios that were modeled in order to develop FBR for infants solely on community-based infant foods. The use of the OPTIFOOD software linear programming analyses used in this study has been described in earlier studies [23,24,25,26,27].

OPTIFOOD Module 1 was performed on the data for each scenario to make sure that the model parameters were yielding realistic diets with energy contents within a range that was large enough to support modeling. For each of the three scenarios, Module 2 (to find food-based recommendations) was performed in order to create two optimum diets, the food pattern diet (i.e., CIF) and the no-food pattern diet (CIF plus underutilized food combinations). The diet with the fewest RNIs departing from the typical meal pattern (CIF) while still adhering to lower and upper food group restrictions was the no-food pattern diet. The focus group discussions confirmed the selected food items for the target group (infants on only CIF, excluding family foods). Module 2 was used to select the most nutrient-rich foods (from the underused food item list) that were likely to the increase nutritional adequacy of CIF and would be required to be evaluated in Module 3. Nutrient-dense foods were defined as those that supplied at least 5% of any one of the nutrients [23,24,25,28].

Diets were modeled for all three scenarios in Module 3, which tests different sets of FBRs and chooses the optimal dietary recommendations for the target population. Per nutrient, seven and twelve seven-day diets were simulated. When modeling the various scenario diets, the cost of food items was taken into consideration. A no-recommendation diet was generated for each scenario in Step (i) of Module 3 to identify problematic nutrients. In the best-case scenario (maximized diet) of the Module 3 diet simulated without FBR limitations, problem nutrients were classified as nutrients that were less than 100% of the RNI [24,25,28]. Underutilized foods were examined separately in Step (ii) of Module 3 before being combined in Step (iii). The final FBRs per scenario were chosen based on the combination of foods that covered 70% of the RNI in the worst case scenario for the majority of nutrients, minimizing the deviation from the local food pattern (CIF), in Step (ii) of Module 3. Underutilized foods were tested individually in Step (ii) of Module 3 and combined in Step (iii) of Module 3. The three scenarios’ final FBRs were contrasted. Table 1 shows the general layout of our methodology for the modules for each scenario.

## 3. Results

### Community-Based Infant Foods

Table 2 shows the list of community-based infant foods. Millet dough and corn dough porridges were the commonly consumed porridges at the household level. A composite blend (Weanimix) was the mainstream recommended blend of maize, peanut, and soybean for infant and young child feeding (IYCF) in Ghana, which is often purchased (few households prepare their own Weanimix).

The sensory attributes of the enriched recipes are summarized in Table 3, Table 4 and Table 5. All recipes in the respective communities were overwhelmingly preferred when compared to the existing CIFs (millet and corn dough porridges) in terms of appearance, aroma, sweetness, texture, overall degree of acceptance and appropriateness of recipes for infant feeding.

Table 6 and Table 7 indicates the nutrient contribution of community-based infant foods and the enriched recipes. Table 6 indicates the nutrient contribution (% RNI) of CIFs (corn or millet porridges) for seven 7-day diet simulations for all the three scenarios. The food pattern diets (CIFs) had three nutrients with % RNI ≥70 and five nutrients with % RNI ≥ 50.

For the no-restriction diet modeled in Scenario 2, all combinations improved the % RNI of CIFs. Using CIFs as a base, five nutrients with % RNI ≥ 70 were observed for pawpaw and soybean combinations. OFSP (seven nutrients) and moringa (eight nutrients) combinations had the highest number of nutrients with % RNI ≥ 50. The best diet in terms of cost and % RNI was the soybean combination diets followed by the pawpaw combination diets. In Scenario 3, all combinations improved the % RNI of all nutrients for CIFs. Using CIFs as a base, six nutrients with % RNI ≥ 70 were observed for moringa–pawpaw, moringa–soybean, and pawpaw–soybean combinations. All combinations had at least seven nutrients with % RNI ≥ 50. The best diets that combine a low cost and high number of nutrients meeting % RNI cut-off points were the moringa–soybean and pawpaw–soybean combinations, respectively, for corn and millet porridges.

Table 7 indicates the nutrient contribution (% RNI) of CIFs for twelve 7-day diet simulations for all the three scenarios. The food pattern diets (CIFs) had three nutrients with % RNI ≥ 70 and five nutrients with % RNI ≥ 50. For the no-restriction diet (Scenario 2), all combinations improved the % RNI of all nutrients for CIFs. Using CIFs as a base, five nutrients with % RNI ≥ 70 were observed for OFSP and pawpaw combinations for corn dough porridges. Six, six, and seven nutrients with % RNI ≥ 70 were observed for OFSP, pawpaw, and moringa combinations, respectively, for millet porridges. OFSP–pawpaw–moringa–soybean combinations had at least seven nutrients with % RNI ≥ 50. The best diets in terms of low costs and higher numbers of micronutrients meeting the % RNI cut-off points was the OFSP and moringa combinations, respectively, for maize and millet dough porridges. In Scenario 3, all combinations improved the % RNI of all nutrients for CIFs. Using CIFs as a base, all combinations had at least six nutrients with % RNI ≥ 70. With the exception of Bambara bean–pawpaw and cowpea–pawpaw combinations which had seven nutrients, all other combinations had at least eight nutrients with % RNI ≥ 50. The best diet in terms of a lower cost and the number of diets with more nutrients meeting the % RNI cut-off levels was the moringa–soybean combinations followed by moringa–pawpaw combinations.

## 4. Discussion

Homemade or CIFs typically contain ingredients that are fresh, tasty, and nourishing for young children [4], especially in nations with rules for the preparation of homemade infant food. Ghana, however, lacks national rules for the preparation of homemade infant food, despite the fact that they exist in several other nations [5,6,7]. The lack of regulations makes it challenging for extension personnel to provide better guidance, but the driving constraints of poverty and lack of knowledge can be tackled through promoting the use of underutilized nutritious foods that are relatively easy to produce. The complementary feeding period is a crucial time in which to introduce infants to novel flavors and textures that will subsequently influence their preferences and acceptance of a wide range of well-known foods [9,10,11].

### 4.1. Nutrient Contribution

The results of our study demonstrate that existing community-based infant foods do not meet the desirable RNI level of all the nutrients under consideration. Incorporating selected underutilized food crops into recipes could improve the percentage RNI contributions of all nutrients of community-based infant foods. The underutilized food crops examined (orange-fleshed sweet potato, moringa, pawpaw, cowpeas, Bambara beans and soybeans) may, however, not be able to cover the nutrient requirement for fat, zinc, and vitamin B-12. An earlier study indicated that younger children aged 6–12 months in Ghana were more likely to depend entirely on infant foods which were inadequate at meeting the nutrient requirements of the infants [29]. In agreement with our study, Boateng et al. (2019) also demonstrated a modest increase in iron and vitamin A amounts in a food study trial using moringa leaves and legumes as fortifiers of infant foods in Ghana [30]. Per this study’s observations, infants that exclusively consume CIFs (even enriched CIFs per this study) in addition to breastmilk may not meet the recommended intakes for fat, zinc, and vitamin B12. As such, alternative strategies should be explored, including the promotion of animal-source foods that are known for providing B12 and zinc in the diet.

Although all the recipe combinations improved the nutrient content of the CIFs in our study, moringa, pawpaw, OFSP, cowpea or soybean recipe combinations were the best, containing 8–10 micronutrients with % RNI ≥ 70 in the twelve 7-day diet simulations. A detailed examination of all the simulations indicated that the enriched recipes had at least four and at most twelve nutrients with % RNI ≥ 50 based on the recipe combinations and simulations. As the number of food items per recipe increases, the number of micronutrients in the % RNI increases. This finding suggest that increasing variety in CIFs, as demonstrated, improves the nutrient intake of infants. We acknowledge, however, that more complex recipes may increase the time required of caregivers to prepare the complementary food.

In terms of the affordability of the various recipes, it was noticed that moringa, pawpaw, and soybeans often appeared in the best diet formulations as the relative cost of their inclusion per key nutrient provided was lower than that of other items. To the best our knowledge, this is the first study to explore the cost of enriched CIFs using these particular foods.

### 4.2. Sensory Assessment

The findings indicate that the underutilized crops recipe were all deemed suitable and appropriate for infant feeding by mothers. The practicality of introducing a food product to a target audience and its acceptance are both predicted by sensory evaluations of the food product [31]. In order to establish a proper socio-cultural framework for the introduction of the enriched infant food recipes, maternal approval was essential [32]. In line with some other studies [31,32], the sensory evaluation in this study explored whether the enriched infant food recipes were more or less acceptable than the widely used community-based infant foods were. This is due to the fact that enriched infant food recipes are novel CFs in the research setting and, as a result, call for comparison to commonly used community-based infant foods.

Overall acceptability was highest for the porridges from the underutilized OFSP, pawpaw, and legumes, possibly as a result of the products’ well-known tastes, fragrances, and appearances [32]. This study’s findings are in line with those of other studies, which discovered that infant foods enriched with OFSP and legumes had high overall acceptance scores [33]. It is interesting to note that while moringa recipe approval was high when compared to that of currently available CIFs, it was somewhat low when compared to that of OFSP and legume recipes, most likely due to its taste. This acceptance level is similar to that of an earlier finding [34]. With repeated exposure and the development of a positive narrative surrounding the intake of green leafy vegetables, moringa being a super food, the acceptance of the moringa recipe may increase or become on par with the that of the OFSP/pawpaw/legume recipes.

The results of this study demonstrate that the enhanced recipes were regarded as suitable and acceptable alternative infant foods and seem to be a potential route to increasing the variety of infant food intake in order to improve nutritional status.

### 4.3. Limitations

We recognize certain significant restrictions on this study. First, we used secondary data rather than measuring the amount currently consumed by infants. In addition, the RNIs were determined through the use of nutritional composition tables that do not fully account for the bioavailability of a nutrient within a given food matrix. Thus, it is likely that while we are sure that the percentage of nutrients attaining the cut-off points was reached in most cases, there is a level of uncertainty about these predicted values due to the lack of bioavailability adjustment.

### 4.4. Conclusions

Community-based infant foods (CIFs) were mainly made of either maize or millet in Northern Ghana. Per our study, infants that exclusively consume CIFs in addition to breastmilk fail to meet the recommended intakes for the nutrients studied, and the inclusion of underused crops can significantly affect nutritional adequacy. Enriched CIFs with underutilized foods (moringa, OFSP, and legumes recipes) provided the right caloric amounts and modest improvement of micronutrient content for infants. According to mothers, these new recipes are suitable and acceptable for feeding infants in Northern Ghana. Future research is necessary to assess the effectiveness of the new recipes at promoting linear growth and improving micronutrient status during the complementary feeding period in the research settings.

## Figures and Tables

**Table 1:** Optifood analysis representation.

Steps	Scenario		
	Community-Based Infant Foods Only	CIFs + One Underutilized Food Item	CIFs + Two Underutilized Food Items
1	Module 1: screen diets for model parameters to ensure Optifood generates realistic diets	Module 1: screen diets for model parameters to ensure Optifood generates realistic diets	Module 1: Screen diets for model parameters to ensure Optifood generates realistic diets
2	Module 2: identify draft recommendations for CIFs only	Module 2: identify draft recommendations for CIFs + one underutilized food item	Module 2: identify draft recommendations for CIFs + two underutilized food items
3	Module 3: test food-based recommendation for CIFs only	Module 3: test food-based recommendation for CIFs + one underutilized food item	Module 3: test food-based recommendation for CIFs + two underutilized food items

CIFs: Community-based infant foods.

**Table 2:** Community-based infant foods in Northern Ghana.

Infant Food	Community		
	Kpulinyin	Dinyanpalgu	Bongbini
Millet dough porridge	X		XX
Corn dough porridge	XX	XX	XX
Composite blend			X

XX: Food prepared by 50% or more households, X: food prepared by few households.

**Table 3:** Paired preference testing—community-based infant food in Kumbungu district and improved predeveloped recipes.

Porridge Characteristics	Kpulinyin Community			
	Group 1		Group 2	
	OFSP_CD	CD	OFSP_CD	CD
	*n* (%)	*n* (%)	*n* (%)	*n* (%)
Appearance	7 (100)	0 (0)	8 (100)	0 (0)
Aroma	7 (100)	0 (0)	8 (100)	0 (0)
Sweetness	7 (100)	0 (0)	8 (100)	0 (0)
Texture	7 (100)	0 (0)	8 (100)	0 (0)
Overall degree of acceptability	7 (100)	0 (0)	8 (100)	0 (0)
Suitability/appropriateness for infants	7 (100)	0 (0)	8 (100)	0 (0)
	PAWPAW_CD	CD	PAWPAW_CD	CD
	*n* (%)	*n* (%)	*n* (%)	*n* (%)
Appearance	7 (100)	0 (0)	8 (100)	0 (0)
Aroma	7 (100)	0 (0)	8 (100)	0 (0)
Sweetness	7 (100)	0 (0)	8 (100)	0 (0)
Texture	7 (100)	0 (0)	8 (100)	0 (0)
Overall degree of acceptability	7 (100)	0 (0)	8 (100)	0 (0)
Suitability/appropriateness for infants	7 (100)	0 (0)	8 (100)	0 (0)
	COWPEA_CD	CD	COWPEA_CD	CD
	*n* (%)	*n* (%)	*n* (%)	*n* (%)
Appearance	7 (100)	0 (0)	8 (100)	0 (0)
Aroma	7 (100)	0 (0)	5 (71)	2 (29)
Sweetness	7 (100)	0 (0)	8 (100)	0 (0)
Texture	7 (100)	0 (0)	8 (100)	0 (0)
Overall degree of acceptability	7 (100)	0 (0)	8 (100)	0 (0)
Suitability/appropriateness for infants	7 (100)	0 (0)	8 (100)	0 (0)
	GN_CD	CD	GN_CD	CD
	*n* (%)	*n* (%)	*n* (%)	*n* (%)
Appearance	10 (100)	0 (0)	10 (100)	0 (0)
Aroma	10 (100)	0 (0)	10 (100)	0 (0)
Sweetness	10 (100)	0 (0)	10 (100)	0 (0)
Texture	10 (100)	0 (0)	10 (100)	0 (0)
Overall degree of acceptability	10 (100)	0 (0)	10 (100)	0 (0)
Suitability/appropriateness for infants	10 (100)	0 (0)	10 (100)	0 (0)
	BAMBARA_CD	CD	BAMBARA_CD	CD
	*n* (%)	*n* (%)	*n* (%)	*n* (%)
Appearance	7 (100)	0 (0)	8 (100)	0 (0)
Aroma	7 (100)	0 (0)	5 (71)	2 (29)
Sweetness	7 (100)	0 (0)	8 (100)	0 (0)
Texture	7 (100)	0 (0)	8 (100)	0 (0)
Overall degree of acceptability	7 (100)	0 (0)	8 (100)	0 (0)
Suitability/appropriateness for infants	7 (100)	0 (0)	8 (100)	0 (0)
	SOYA_CD	CD	SOYA_CD	CD
	*n* (%)	*n* (%)	*n* (%)	*n* (%)
Appearance	7 (100)	0 (0)	8 (100)	0 (0)
Aroma	7 (100)	0 (0)	8 (100)	0 (0)
Sweetness	7 (100)	0 (0)	8 (100)	0 (0)
Texture	7 (100)	0 (0)	8 (100)	0 (0)
Overall degree of acceptability	7 (100)	0 (0)	8 (100)	0 (0)
Suitability/appropriateness for infants	7 (100)	0 (0)	8 (100)	0 (0)
	MORINGA_CD	CD	MORINGA_CD	CD
	*n* (%)	*n* (%)	*n* (%)	*n* (%)
Appearance	0 (0)	7 (100)	8 (100)	0 (0)
Aroma	7 (100)	0 (0)	8 (100)	0 (0)
Sweetness	0 (0)	7 (100)	8 (100)	0 (0)
Texture	0 (0)	7 (100)	8 (100)	0 (0)
Overall degree of acceptability	0 (0)	7 (100)	8 (100)	0 (0)
Suitability/appropriateness for infants	0 (0)	7 (100)	8 (100)	0 (0)

CD = corn dough porridge, OFSP_CD = OFSP and corn dough porridge, GN_CD = groundnut and corn dough porridge, COWPEA_CD = cowpea and corn dough porridge, BAMBARA_CD = Bambara beans and corn dough porridge, SOYA_CD = soya beans and corn dough porridge, MORINGA_CD = moringa and corn dough porridge, PAWPAW_CD = pawpaw and corn dough porridge.

**Table 4:** Paired preference testing—community-based infant food in Kumbungu district and improved predeveloped recipes.

Porridge Characteristics	Dinyanpalgu Community			
	Group 1		Group 2	
	OFSP_CD	CD	OFSP_CD	CD
	*n* (%)	*n* (%)	*n* (%)	*n* (%)
Appearance	7 (100)	0 (0)	8 (100)	0 (0)
Aroma	7 (100)	0 (0)	8 (100)	0 (0)
Sweetness	7 (100)	0 (0)	8 (100)	0 (0)
Texture	7 (100)	0 (0)	8 (100)	0 (0)
Overall degree of acceptability	7 (100)	0 (0)	8 (100)	0 (0)
Suitability/appropriateness for infants	7 (100)	0 (0)	8 (100)	0 (0)
	PAWPAW_CD	CD	PAWPAW_CD	CD
	*n* (%)	*n* (%)	*n* (%)	*n* (%)
Appearance	7 (100)	0 (0)	8 (100)	0 (0)
Aroma	7 (100)	0 (0)	8 (100)	0 (0)
Sweetness	7 (100)	0 (0)	8 (100)	0 (0)
Texture	7 (100)	0 (0)	8 (100)	0 (0)
Overall degree of acceptability	7 (100)	0 (0)	8 (100)	0 (0)
Suitability/appropriateness for infants	7 (100)	0 (0)	8 (100)	0 (0)
	COWPEA_CD	CD	COWPEA_CD	CD
	*n* (%)	*n* (%)	*n* (%)	*n* (%)
Appearance	7 (100)	0 (0)	8 (100)	0 (0)
Aroma	7 (100)	0 (0)	5 (71)	2 (29)
Sweetness	7 (100)	0 (0)	8 (100)	0 (0)
Texture	7 (100)	0 (0)	8 (100)	0 (0)
Overall degree of acceptability	7 (100)	0 (0)	8 (100)	0 (0)
Suitability/appropriateness for infants	7 (100)	0 (0)	8 (100)	0 (0)
	GN_CD	CD	GN_CD	CD
	*n* (%)	*n* (%)	*n* (%)	*n* (%)
Appearance	10 (100)	0 (0)	10 (100)	0 (0)
Aroma	10 (100)	0 (0)	10 (100)	0 (0)
Sweetness	10 (100)	0 (0)	10 (100)	0 (0)
Texture	10 (100)	0 (0)	10 (100)	0 (0)
Overall degree of acceptability	10 (100)	0 (0)	10 (100)	0 (0)
Suitability/appropriateness for infants	10 (100)	0 (0)	10 (100)	0 (0)
	BAMBARA_CD	CD	BAMBARA_CD	CD
	*n* (%)	*n* (%)	*n* (%)	*n* (%)
Appearance	7 (100)	0 (0)	8 (100)	0 (0)
Aroma	7 (100)	0 (0)	5 (71)	2 (29)
Sweetness	7 (100)	0 (0)	8 (100)	0 (0)
Texture	7 (100)	0 (0)	8 (100)	0 (0)
Overall degree of acceptability	7 (100)	0 (0)	8 (100)	0 (0)
Suitability/appropriateness for infants	7 (100)	0 (0)	8 (100)	0 (0)
	SOYA_CD	CD	SOYA_CD	CD
	*n* (%)	*n* (%)	*n* (%)	*n* (%)
Appearance	7 (100)	0 (0)	8 (100)	0 (0)
Aroma	7 (100)	0 (0)	8 (100)	0 (0)
Sweetness	7 (100)	0 (0)	8 (100)	0 (0)
Texture	7 (100)	0 (0)	8 (100)	0 (0)
Overall degree of acceptability	7 (100)	0 (0)	8 (100)	0 (0)
Suitability/appropriateness for infants	7 (100)	0 (0)	8 (100)	0 (0)
	MORINGA_CD	CD	MORINGA_CD	CD
	*n* (%)	*n* (%)	*n* (%)	*n* (%)
Appearance	0 (0)	7 (100)	8 (100)	0 (0)
Aroma	7 (100)	0 (0)	8 (100)	0 (0)
Sweetness	0 (0)	7 (100)	8 (100)	0 (0)
Texture	0 (0)	7 (100)	8 (100)	0 (0)
Overall degree of acceptability	0 (0)	7 (100)	8 (100)	0 (0)
Suitability/appropriateness for infants	0 (0)	7 (100)	8 (100)	0 (0)

CD = corn dough porridge, OFSP_CD = OFSP and corn dough porridge, GN_CD = groundnut and corn dough porridge, COWPEA_CD = cowpea and corn dough porridge, BAMBARA_CD = Bambara beans and corn dough porridge, SOYA_CD = soya beans and corn dough porridge, MORINGA_CD = moringa and corn dough porridge, PAWPAW_CD = pawpaw and corn dough porridge.

**Table 5:** Paired preference testing—community-based infant food in East Manprusi district and improved predeveloped recipes.

Porridge Characteristics	Bongbini Community							
	Group 1		Group 2		Group 1		Group 2	
	OFSP_MILLET *n* (%)	MD *n* (%)	OFSP_MILLET *n* (%)	MD *n* (%)	OFSP_CD *n* (%)	CD *n* (%)	OFSP_CD *n* (%)	CD *n* (%)
Appearance	6 (100)	0 (0)	8 (100)	0 (0)	6 (100)	0 (0)	8 (100)	0 (0)
Aroma	6 (100)	0 (0)	8 (100)	0 (0)	6 (100)	0 (0)	8 (100)	0 (0)
Sweetness	6 (100)	0 (0)	8 (100)	0 (0)	6 (100)	0 (0)	8 (100)	0 (0)
Texture	6 (100)	0 (0)	8 (100)	0 (0)	6 (100)	0 (0)	8 (100)	0 (0)
Overall degree of acceptability	6 (100)	0 (0)	8 (100)	0 (0)	6 (100)	0 (0)	8 (100)	0 (0)
Suitability/appropriateness for infants	6 (100)	0 (0)	8 (100)	0 (0)	6 (100)	0 (0)	8 (100)	0 (0)
	PAWPAW_MILLET *n* (%)	MD *n* (%)	PAWPAW_MILLET *n* (%)	MD *n* (%)	PAWPAW_CD *n* (%)	CD *n* (%)	PAWPAW_CD *n* (%)	CD *n* (%)
Appearance	6 (100)	0 (0)	8 (100)	0 (0)	6 (100)	0 (0)	8 (100)	0 (0)
Aroma	6 (100)	0 (0)	8 (100)	0 (0)	6 (100)	0 (0)	8 (100)	0 (0)
Sweetness	6 (100)	0 (0)	8 (100)	0 (0)	6 (100)	0 (0)	8 (100)	0 (0)
Texture	6 (100)	0 (0)	8 (100)	0 (0)	6 (100)	0 (0)	8 (100)	0 (0)
Overall degree of acceptability	6 (100)	0 (0)	8 (100)	0 (0)	6 (100)	0 (0)	8 (100)	0 (0)
Suitability/appropriateness for infants	6 (100)	0 (0)	8 (100)	0 (0)	6 (100)	0 (0)	8 (100)	0 (0)
	COWPEA_MILLET *n* (%)	MD *n* (%)	COWPEA_MILLET *n* (%)	MD *n* (%)	COWPEA_CD *n* (%)	CD *n* (%)	COWPEA_CD *n* (%)	CD *n* (%)
Appearance	6 (100)	0 (0)	8 (100)	0 (0)	6 (100)	0 (0)	8 (100)	0 (0)
Aroma	6 (100)	0 (0)	8 (100)	0 (0)	6 (100)	0 (0)	8 (100)	0 (0)
Sweetness	6 (100)	0 (0)	8 (100)	0 (0)	6 (100)	0 (0)	8 (100)	0 (0)
Texture	6 (100)	0 (0)	8 (100)	0 (0)	6 (100)	0 (0)	8 (100)	0 (0)
Overall degree of acceptability	6 (100)	0 (0)	8 (100)	0 (0)	6 (100)	0 (0)	8 (100)	0 (0)
Suitability/appropriateness for infants	6 (100)	0 (0)	8 (100)	0 (0)	6 (100)	0 (0)	8 (100)	0 (0)
	BAMBARA_MILLET *n* (%)	MD *n* (%)	BAMBARA_MILLET *n* (%)	MD *n* (%)	BAMBARA_CD *n* (%)	CD *n* (%)	BAMBARA_CD *n* (%)	CD *n* (%)
Appearance	6 (100)	0 (0)	4 (50)	4 (50)	6 (100)	0 (0)	6 (75)	2 (25)
Aroma	6 (100)	0 (0)	4 (50)	4 (50)	6 (100)	0 (0)	8 (100)	0 (0)
Sweetness	6 (100)	0 (0)	4 (50)	4 (50)	6 (100)	0 (0)	6 (75)	2 (25)
Texture	6 (100)	0 (0)	4 (50)	4 (50)	6 (100)	0 (0)	8 (100)	0 (0)
Overall degree of acceptability	6 (100)	0 (0)	4 (50)	4 (50)	6 (100)	0 (0)	6 (75)	2 (25)
Suitability/appropriateness for infants	6 (100)	0 (0)	4 (50)	4 (50)	6 (100)	0 (0)	6 (75)	2 (25)
	SOYA_MILLET *n* (%)	MD *n* (%)	SOYA_MILLET *n* (%)	MD *n* (%)	SOYA_CD *n* (%)	CD *n* (%)	SOYA_CD *n* (%)	CD *n* (%)
Appearance	6 (100)	0 (0)	8 (100)	0 (0)	6 (100)	0 (0)	8 (100)	0 (0)
Aroma	6 (100)	0 (0)	8 (100)	0 (0)	6 (100)	0 (0)	8 (100)	0 (0)
Sweetness	6 (100)	0 (0)	8 (100)	0 (0)	6 (100)	0 (0)	8 (100)	0 (0)
Texture	6 (100)	0 (0)	8 (100)	0 (0)	6 (100)	0 (0)	8 (100)	0 (0)
Overall degree of acceptability	6 (100)	0 (0)	8 (100)	0 (0)	6 (100)	0 (0)	8 (100)	0 (0)
Suitability/appropriateness for infants	6 (100)	0 (0)	8 (100)	0 (0)	6 (100)	0 (0)	8 (100)	0 (0)
	MORINGA_MILLET *n* (%)	MD *n* (%)	MORINGA_MILLET *n* (%)	MD *n* (%)	MORINGA_CD *n* (%)	CD *n* (%)	MORINGA_CD *n* (%)	CD *n* (%)
Appearance	4 (67)	2 (33)	8 (100)	0 (0)	6 (100)	0 (0)	8 (100)	0 (0)
Aroma	6 (100)	0 (0)	8 (100)	0 (0)	6 (100)	0 (0)	8 (100)	0 (0)
Sweetness	6 (10)	0 (0)	8 (100)	0 (0)	6 (100)	0 (0)	8 (100)	0 (0)
Texture	6 (100)	0 (0)	8 (100)	0 (0)	6 (100)	0 (0)	8 (100)	0 (0)
Overall degree of acceptability	6 (100)	0 (0)	8 (100)	0 (0)	6 (100)	0 (0)	8 (100)	0 (0)
Suitability/appropriateness for infants	6 (100)	0 (0)	8 (100)	0 (0)	6 (100)	0 (0)	8 (100)	0 (0)

CD = corn dough porridge, MD = millet dough porridge, OFSP_CD = OFSP and corn dough porridge, OFSP_MILLET = OFSP and millet porridge, Cowpea_MILLET = cowpea and millet porridge, COWPEA_CD = cowpea and corn dough porridge, BAMBARA_MILLET = Bambara beans and millet porridge, BAMBARA_CD = Bambara beans and corn dough porridge, SOYA_MILLET = soya beans and millet porridge, SOYA_CD = soya beans and corn dough porridge, MORINGA_MILLET = moringa and millet porridge, MORINGA_CD = moringa and corn dough porridge, PAWPAW_CD = pawpaw and corn dough porridge, PAWPAW_MILLET = pawpaw and millet dough porridge

**Table 6:** Nutrient contribution of underutilized crops to community-based infant foods—seven 7-day simulations.

Recipe	% RNI													Cost (GhC)	No. of Nut ≥ 70% RNI	No. of Nut ≥ 50% RNI
	Protein	Fat	Calcium	Vit C	Thiamin	Riboflavin	Niacin	Vit B-6	Folate	Vit B-12	Vit A RAE	Iron	Zinc			
CD *	103.6	20	12.1	39.3	117.9	36.9	60.8	122.3	58.2	3	12.2	24	25.5	1	3	5
MD *	100.8	20.8	12.2	39.3	117.8	40.7	60.1	120.7	61.4	3	12.2	31.6	28.4	1.2	3	5
CD–PP	103.6	20	15.3	164.1	117.9	39.3	64.2	122.3	88.6	3.8	23.6	24.8	25.5	1.1	5	6
CD–CP	131.6	20	14.7	40.1	122.8	38.7	64.4	122.3	94.6	3	12.2	28.7	32.4	1.1	4	5
CD–BB	131.4	24.1	16.2	40.1	119	36.9	65.1	130.5	119.7	3	12.4	25.4	26.6	1	4	5
CD–SB	157.1	35.5	24.6	43.3	152.8	73.9	60.8	128.7	144.9	3	12.3	36.5	31.8	1	5	6
CD–OFSP	103.6	20	14.6	60	117.9	38	65.6	125	59.6	3.2	51.7	24	25.9	1	3	7
CD–MO	113.5	20.7	33	83	122	54.6	61.5	161.4	61	3.1	67.2	29.5	26.4	1.2	4	8
MD–PP	100.8	20.8	15.4	164.1	117.8	43.2	63.5	120.7	91.9	3.8	23.6	32.5	28.4	1.3	5	6
MD–CP	129.5	20.8	14.7	40.1	122.4	43	63.7	120.7	97.9	3	12.2	36.5	35.5	1.3	4	5
MD–SB	155	36.3	24.6	43.3	152.4	78.2	60.1	127.2	148.2	3	12.3	44.3	34.8	1.2	5	6
MD–OFSP	100.8	20.8	14.6	60	117.8	42	64.9	123.4	62.9	3.2	51.7	31.6	28.8	1.2	3	7
MD–MO	110.9	21.5	33.1	83	121.8	58.5	60.8	159.8	64.2	3.1	67.2	37.2	29.3	1.4	4	8
MD–BB	129.3	24.9	16.2	40.1	118.7	41.2	64.4	129	123	3	12.4	33.2	29.6	1.2	4	5
CD–PP–CP	131.6	20	17.9	164.9	122.8	41.7	67.8	122.3	125.2	3.9	23.6	30.3	32.4	1.2	5	6
CD–PP–MO	113.5	20.7	36.2	207.8	122	57.1	64.9	161.4	91.5	4	78.6	30.4	26.4	1.4	6	8
CD–PP–OFSP	103.6	20	17.8	184.7	117.9	40.6	69	125	90.2	4.1	63	24.9	25.9	1.1	5	7
CD–PP–BB	131.4	24.1	19.4	164.9	119	40	68.5	130.5	150.3	3.9	23.7	27.1	26.6	1.1	5	6
CD–OFSP–MO	113.5	20.7	35.4	103.7	122	55.8	66.3	164.1	62.5	3.4	106.7	29.5	26.8	1.2	5	8
CD–PP–SB	157.1	35.5	27.8	168	152.8	76.9	64.2	128.7	175.6	3.8	23.6	38.2	31.8	1.2	6	7
CD–CP–MO	141.7	20.7	35.5	83.8	127	56.6	65.1	161.4	97.5	3.2	67.2	34.5	33.4	1.3	5	8
CD–SB–MO	167.5	36.2	45.5	87	157.1	91.8	61.5	168	147.8	3.1	67.3	42.2	32.8	1	6	8
CD–CP–OFSP	131.6	20	17.1	60.7	122.8	40.2	69.2	125	96.2	3.3	51.7	29	32.9	1	4	7
CD–SB–OFSP	157.1	35.5	27.1	63.9	152.8	75.4	65.6	131.7	146.5	3.2	51.8	36.8	32.3	1.1	5	8
CD–BB–OFSP	131.4	24.1	18.6	60.7	119	38.4	69.9	133.5	121.3	3.2	51.9	25.7	27	1.3	4	7
CD–BB–MO	141.8	24.8	37	83.8	123.3	54.8	65.8	169.8	122.6	3.2	67.4	31.1	27.5	1.1	5	8
MD–SB–OFSP	155	36.3	27.1	63.9	152.4	79.8	64.9	130.2	149.8	3.2	51.8	44.6	35.3	1.4	5	8
MD–CP–OFSP	129.5	20.8	17.1	60.7	122.4	44.5	68.6	123.4	99.5	3.2	51.7	36.8	35.9	1.4	4	7
MD–BB–MO	139.3	25.5	37	83.8	123.1	59.2	65.1	168.4	125.9	3.1	67.4	38.9	30.5	1.3	5	8
MD–SB–MO	165	37	45.5	87	156.9	96.2	60.8	166.6	151.1	3.1	67.3	50	35.8	1.4	6	9
MD–CP–MO	139.6	21.5	35.5	83.8	126.6	61	64.5	159.8	100.8	3.2	67.2	42.3	36.4	1.6	5	8
MD–OFSP–MO	110.9	21.5	35.5	103.7	121.8	59.7	65.7	162.6	65.8	3.3	106.7	37.2	29.7	1.3	5	8
MD–PP–BB	129.3	24.9	19.4	164.9	118.7	44.6	67.8	129	153.6	3.9	23.7	34.9	29.6	1.2	5	6
MD–PP–SB	155	36.3	27.8	168	152.4	81.7	63.5	127.2	178.9	3.8	23.6	46.1	34.8	1.2	6	7
MD–PP–CP	129.5	20.8	17.9	164.9	122.4	46.3	67.2	120.7	128.5	3.9	23.6	38.2	35.5	1.5	5	6
PP–MD–OFSP	100.8	20.8	17.8	184.7	117.8	44.7	68.4	123.4	93.5	4	63	32.7	28.8	1.5	5	7
MD–PP–MO	110.9	21.5	36.2	207.8	121.8	61.2	64.3	159.8	94.8	4	78.6	38.1	29.3	1.4	6	8
MD–BB–OFSP	129.3	24.9	18.6	60.7	118.7	42.8	69.2	132	124.6	3.2	51.9	33.5	30	1.3	4	7

* Community-based infant food recipes; CD: corn dough, MD: millet dough, PP: pawpaw, CP: cowpea, BB: Bambara beans, SB: soybeans, OFSP: orange-fleshed sweet potato, MO: moringa; GhC: Ghanaian cedi.

**Table 7:** NuTable—7-day simulations.

Recipe	% RNI													Cost(GhC)	No. of Nut ≥ 70% RNI	No. of Nut ≥ 50% RNI
	Protein	Fat	Calcium	Vit C	Thiamin	Riboflavin	Niancin	Vit B-6	Folate	Vit B-12	Vit A RAE	Iron	Zinc			
CD *	92.7	19.2	12.1	39.3	110.9	36.9	62.7	117.9	60.3	3.4	12.2	24.8	23.4	1.1	3	5
MD *	88.1	20.7	12.3	39.3	111	44.8	61.5	115.2	66.3	3.3	12.2	38.9	28.9	1.4	3	5
CD	92.7	19.2	12.1	39.3	110.9	36.9	62.7	117.9	60.3	3.4	12.2	24.8	23.4	1.1	3	5
CD–PP	92.7	19.2	21.8	413.7	110.9	45	72.9	117.9	152.3	6	46.2	29.4	23.4	1.5	5	6
CD–CP	153.7	19.2	16.5	40.6	129.4	40.7	69	120.3	123.1	3.5	12.2	34.6	37.7	1.3	3	5
CD–BB	156.4	27.2	19.1	40.6	124.7	37.8	70.1	137.1	166.1	3.5	12.5	29	28.1	1.1	4	5
CD–SB	209.3	48.9	35	46.5	188	105.9	62.7	136.1	219.7	3.4	12.3	49.8	38.3	1.1	4	6
CD–OFSP	95	19.5	16.3	74.7	113.6	38.9	71.2	123.8	63.1	3.8	80	25.4	24.7	1.2	5	7
CD–MO	111.5	20.5	50.4	119.5	120.1	69.3	64.1	190	65.6	3.7	113.1	35	25.1	1.6	4	9
MD–PP	88.1	20.7	21.8	413.7	111	53.9	71.7	115.2	158.3	6	46.2	43.9	28.9	1.9	6	7
MD–CP	150.3	20.7	16.5	40.6	129.8	49.6	67.8	118.8	129.1	3.5	12.2	49	43.5	1.6	4	5
MD–BB	153	28.8	19.1	40.6	125	46.7	68.9	135.6	172.1	3.4	12.5	43.4	33.9	1.4	4	5
MD–SB	205.9	50.5	35.1	46.5	188.4	114.8	61.6	134.6	225.7	3.3	12.3	64.2	44.2	1.4	5	8
MD–OFSP	90.8	20.9	16.5	74.7	113.1	46.9	70	121.1	69.1	3.7	80	39.5	30.3	1.5	6	7
MD–MO	106.6	22.1	50.5	119.5	120.3	77.3	62.9	187.3	71.7	3.6	113.1	49.2	30.7	1.5	7	9
CD–PP–MO	111.5	20.5	59.2	462.7	120.1	77.2	73.4	190	149.9	6.1	144.2	39.7	25.1	1.7	8	9
CD–CP–MO	175.6	20.5	54.7	120.8	139	73.8	70.3	194.4	128.4	3.8	113.1	45.3	39.9	1.8	8	9
CD–SB–MO	231.2	50.5	73.3	126.7	197.7	139.1	64.2	210.3	225	3.7	113.2	60.5	40.6	1.5	8	11
CD–BB–MO	178.3	28.8	57.3	120.8	134.3	71	71.5	211.2	171.4	3.8	113.4	39.7	30.3	1.5	8	9
CD–SB–OFSP	210.9	49	39.2	81.9	189.8	108.9	71.2	144	222.4	3.8	80	50.6	40	1.6	8	9
CD–PP–OFSP	95	19.5	25.2	417.9	113.6	47	80.5	123.8	147.4	6.2	111.1	29.8	24.7	1.6	7	7
CD–OFSP–MO	113.8	20.8	54.6	154.9	122.1	71.5	72.5	195.9	68.4	4.1	180.8	35.6	26.6	1.7	7	9
CD–PP–SB	211.1	48.9	44.4	389.7	197.6	116.6	74.9	138.6	305.4	5.8	43.5	56.3	39.2	1.2	7	8
CD–CP–OFSP	155.3	19.5	20.7	76	131.2	43.6	77.5	128.2	125.8	3.9	80	35.5	39.3	1.2	7	7
CD–BB–OFSP	158	27.5	23.3	76	126.5	40.8	78.6	145	168.8	3.9	80.3	29.9	29.7	1.6	7	7
CD–PP–CP	154	19.2	25.4	383.8	133.9	50.5	78.8	120.3	207.4	6	43.4	40.3	37.8	1.8	6	7
CD–PP–BB	157.2	27.2	28.2	383.8	131.8	48.1	80.9	137.9	250.8	5.9	43.7	35.1	28.5	1.4	6	6
MD–SB–MO	227.8	52.2	73.3	126.7	198.4	147.9	63	208.7	231	3.6	113.2	75	46.5	1.9	9	11
MD–PP–MO	106.6	22.1	59.3	462.7	120.3	86.1	72.2	187.3	156	6.1	144.2	54.1	30.7	1.9	8	10
MD–PP–SB	208.6	50.5	44.8	389.7	200.1	126.3	74.9	138.4	312.3	5.8	43.5	71	45.4	1.9	8	9
MD–OFSP–MO	109.2	22.3	54.7	154.9	122.3	79.7	71.3	193.2	74.4	4	180.8	49.9	32.3	2	8	9
MD–BB–MO	174.9	30.4	57.3	120.8	134.7	79.8	70.3	209.7	177.4	3.8	113.4	54.2	36.2	2.3	8	10
MD–SB–OFSP	207.5	50.6	39.3	81.9	190.2	117.8	70.1	142.5	228.5	3.7	80	65.1	45.9	1.9	8	10
MD–PP–OFSP	90.8	20.9	25.2	417.9	113.1	55.9	79.3	121.1	153.4	6.2	111.1	44.2	30.3	1.5	7	8
MD–CP–MO	172.2	22.2	54.8	120.8	139.4	82.6	69.1	192.9	134.4	3.8	113.1	59.8	45.8	1.5	7	10
MD–CP–OFSP	151.9	20.9	20.7	76	131.5	52.4	76.2	126.7	131.9	3.9	80	49.9	45.2	2.1	7	8
MD–BB–OFSP	154.6	29	23.3	76	126.8	49.6	77.4	143.5	174.9	3.8	80.3	44.3	35.6	2.1	7	7
MD–PP–CP	150.8	20.7	25.8	383.8	135.8	59.9	78	118.9	213.5	5.9	43.4	55	43.7	1.9	6	8
MD–PP–BB	154.7	28.8	28.6	383.8	134.3	57.7	80.9	137.8	257.7	5.9	43.7	49.9	34.7	1.7	6	7

* Community-based infant food recipes; CD: corn dough, MD: millet dough, PP: pawpaw, CP: cowpea, BB: Bambara beans, SB: soybeans, OFSP: orange-fleshed sweet potato, MO: moringa; GhC: Ghanaian cedi.

## Data Availability

The data presented in this study are available within this article.

## Supplementary Materials

The following supporting information can be downloaded at https://www.mdpi.com/article/10.3390/nu15112593/s1; SP1: Infant Food Recipes.
