# Supplementary material for: Suitability and Potential Nutrient Contribution of Underutilized Foods in Community-Based Infant Foods in Northern Ghana
Source: Nutrients. 2023 Jun 1;15(11):2593. doi: 10.3390/nu15112593 (PMC10255816; doi:10.3390/nu15112593)
Supplement: Supplementary file 1 [file nutrients-15-02593-s001.zip › nutrients-2376127-supplementary.pdf]

SP 1. Infant Food Recipes

SP 1. Infant Food Recipes

Major ingredients used for trials

- Corn dough
- Millet dough
- Orange-fleshed sweet potato puree (OFSP)
- Pawpaw puree
- Moringa leaves juice/powder
- Soybean puree
- Cowpea puree
- Bambara bean puree
- Peanut paste
- Grinded boiled peanut

Preparation of corn dough

- Soak corn in water overnight, strain water out and mill
- Add a little water and mix to form dough

Preparation of millet dough

- Soak millet overnight and mill without straining water out
- Leave to settle down
- Separate millet water from dough any time you want to prepare porridge

Preparation of orange-fleshed sweetpotato (OFSP) puree

- Wash and boil OFSP roots
- Strain water and leave to cool and remove skin
- Add strained water to peeled boiled roots and mash or blend into puree

Preparation of pawpaw puree

- Peel pawpaw and mash or blend with little water

Preparation of moringa juice

- Steam moringa for 5 minutes
- Grind and squeeze out juice

Preparation of soybean puree

- Soak, dehull and blend/grind into puree

Preparation of cowpea/Bambara bean puree

- Boil beans until soft
- Blend or mash

Preparation of peanut paste

- Roast and remove the skin
- Mill to form smooth paste

Preparation of grinded boiled peanut

- Boil peanuts and allow to cool
- Grinded peanut

Time of all recipe preparation

- All recipes take from 7 – 11 mins of preparation.

Handy measure

- 1 household soup ladle is approximately 68 g.

Source of heat

- Liquified petroleum gas/charcoal/fire wood

Abbreviations:

tbsp – tablespoon

tsp – teaspoon

RECIPES

1. **Corn/OFSP**

   Ingredients
   - 3 tbsp. OFSP puree
   - 1 ladle corn dough
   - 3 ladles water
   - A pinch of salt
   - 1 tbsp. vegetable oil

   Method
   - Heat oil
   - Add 2 ladles of water and boil.
   - Add OFSP puree and stir.
   - Add salt to corn dough and mix with water until smooth paste.
   - Add to OFSP mixture on fire.
   - Stir till it thickens to form porridge.
   - Leave to simmer for 3 mins.

2. **Corn /Pawpaw**

   Ingredients
   - 1 ladle corn dough
   - 7 Tbs pawpaw puree
   - 3 ladles water
   - A pinch of salt
   - 1 tbsp. vegetable oil

   Method
   - Heat oil
   - Add 2 ladles of water and boil.
   - Add pawpaw and stir.
   - Add salt to corn dough and mix with remaining water until smooth paste.
   - Add to pawpaw on fire.
   - Stir till it thickens to form porridge.
   - Leave to simmer for 3 mins.

3. **Corn /Moringa**

   Ingredients
   - 1 ladle corn dough
   - 3 ladles water
   - A pinch of salt
   - 1 tbsp. vegetable oil
   - 1 tbsp. moringa juice

   Method
   - Heat oil
   - Add 2 ladles of water and boil.
   - Add salt to corn dough and mix with remaining water until smooth paste.
   - Add paste to boiling water
   - Stir till it thickens to form porridge.

- Add moringa juice.
- Leave to simmer for 3 mins.

4. **Corn/Cowpea**

Ingredients

- 1 ladle corn dough
- 3 Tbs cowpea puree
- 3 ladles water
- A pinch of salt.
- 1 tbsp. vegetable oil

Method

- Heat oil
- Add 2 ladles of water and boil.
- Add cowpea puree and stir.
- Add salt to corn dough and mix with remaining water until smooth paste.
- Add to mixture on fire.
- Stir till it thickens to form porridge.
- Leave to simmer for 3 mins.

5. **Corn/Bambara beans**

Ingredients

- 3 ladles water
- 3 tbsp. Bambara bean puree
- 1 ladle corn dough
- 1 tbsp. vegetable oil

Method

- Heat oil
- Boil 2 ladles of water.
- Add Bambara beans puree to boiling water.
- Mix remaining water with corn dough to form paste, add salt.
- Add paste to mixture on fire and stir to form porridge.
- Leave to simmer for 2 mins.

6. **Corn/Soybean**

Ingredients

- 3 ladles water
- 3 Tbs soybean puree
- 1 ladle corn dough
- 1 tbsp. vegetable oil

Method

- Heat oil
- Boil 2 ladles of water.
- Add soybean puree to boiling water and stir until it thickens.
- Mix remaining water with corn dough to form paste, add salt.
- Add paste to mixture on fire and stir to form porridge.
- Leave to simmer for 2 mins.

7. **Corn /Peanut paste**

Ingredients

- 3 tbsp. of peanut paste
- 1 ladle corn dough
- 3 ladles water

Method

- Pour 2 ladles of water in pot and boil.
- Add peanut paste and stir.
- Mix corn dough with water until smooth paste.
- Add to peanut mixture on fire.
- Stir till it thickens to form porridge.
- Leave to simmer for 3 mins.

8. **Corn /Pawpaw/Moringa**

Ingredients

- 1 ladle corn dough
- 7 tbsp. pawpaw puree
- 3 ladles water
- A pinch of salt
- 1 tbsp. vegetable oil
- A pinch of moringa powder or 1 tbsp. moringa juice

Method

- Heat oil
- Add 2 ladles of water and boil.
- Add pawpaw and stir.
- Add salt to corn dough and mix with remaining water until smooth paste.
- Add to pawpaw on fire.
- Stir till it thickens to form porridge.
- Add moringa.
- Leave to simmer for 3 mins.

9. **Corn/Pawpaw/OFSP**

Ingredients

- 1 ladle corn dough
- 4 tbsp. OFSP puree
- 3 tbsp. pawpaw puree
- 3 ladles of water
- A pinch of salt
- Heat oil

Method

- Heat oil
- Add 2 ladles of water and boil.
- Add OFSP and pawpaw purees and stir.
- Add salt to corn dough and mix with remaining water until smooth paste.
- Add to mixture on fire.
- Stir till it thickens to form porridge.
- Leave to simmer for 3 mins.

10. **Corn/Pawpaw/Cowpea**

Ingredients

Ingredients

- 1 ladle corn dough
- 3 tbsp. pawpaw puree
- 2 tbsp. cowpea puree
- 3 ladles water
- A pinch of salt.
- 1 tbsp. vegetable oil

Method

- Heat oil
- Add 2 ladles of water and boil.
- Add pawpaw and cowpea purees and stir.
- Add salt to corn dough and mix with remaining water until smooth paste.
- Add to mixture on fire.
- Stir till it thickens to form porridge.
- Leave to simmer for 3 mins.

11. Corn/Pawpaw/Bambara beans

Ingredients

- 3 ladles water
- 4 tbsp. Bambara bean puree
- 3 tbsp. pawpaw puree
- 1 ladle corn dough
- 1 tbsp. vegetable oil

Method

- Heat oil
- Boil 2 ladles of water.
- Add pawpaw puree to boiling water.
- Mix remaining water with corn dough to form paste, add salt.
- Add paste to mixture on fire and stir to form porridge.
- Add Bambara beans to porridge and mix.
- Leave to simmer for 2 mins.

12. Corn/ Pawpaw/Soy beans

Ingredients

- 1 ladle corn dough
- 4 tbsp. pawpaw puree
- 2 tbsp. soy bean puree
- 3 ladles water
- A pinch of salt.
- 1 tbsp. vegetable oil

Method

- Heat oil
- Add 2 ladles of water and boil.
- Add pawpaw and soy bean purees and stir.
- Add salt to corn dough and mix with remaining water until smooth paste.
- Add to mixture on fire.
- Stir till it thickens to form porridge.
- Leave to simmer for 3 mins.

13. Corn/OFSP/Moringa

Ingredients

- 1 ladle corn dough
- 4 tbsp. OFSP puree
- 3 ladles water
- 1 tbs moringa juice
- A pinch of salt.
- 1 tbsp. vegetable oil

Method

- Heat oil
- Add 2 ladles of water and boil.
- Add OFSP purees and stir.
- Add salt to corn dough and mix with remaining water until smooth paste.
- Add to mixture on fire.
- Stir till it thickens to form porridge.
- Add moringa juice to porridge and mix.
- Leave to simmer for 3 mins.

14. Corn/OFSP/Cowpea

Ingredients

- 1 ladle corn dough
- 4 tbsp. OFSP puree
- 2 tbsp. cowpea puree
- 3 ladles water
- A pinch of salt.
- 1 tbsp. vegetable oil

Method

- Heat oil
- Add 2 ladles of water and boil.
- Add OFSP and cowpea purees and stir.
- Add salt to corn dough and mix with remaining water until smooth paste.
- Add to mixture on fire.
- Stir till it thickens to form porridge.
- Leave to simmer for 3 mins.

15. Corn/OFSP/Bambara beans

Ingredients

- 1 ladle corn dough
- 4 tbsp. OFSP puree
- 2 tbsp. Bambara beans puree
- 3 ladles water
- A pinch of salt.
- 1 tbsp. vegetable oil

Method

- Heat oil
- Add 2 ladles of water and boil.
- Add OFSP and Bambara beans purees and stir.
- Add salt to corn dough and mix with remaining water until smooth paste.
- Add to mixture on fire.
- Stir till it thickens to form porridge.
- Leave to simmer for 3 mins.

16. Corn/OFSP/Soy beans

Ingredients

- 1 ladle corn dough
- 4 tbsp. OFSP puree
- 2 tbsp. soy bean puree
- 3 ladles water
- A pinch of salt.
- 1 tbsp. vegetable oil

Method

- Heat oil
- Add 2 ladles of water and boil.
- Add OFSP and soy bean purees and stir.
- Add salt to corn dough and mix with remaining water until smooth paste.
- Add to mixture on fire.
- Stir till it thickens to form porridge.
- Leave to simmer for 3 mins.

17. Corn/Soy beans/Moringa

Ingredients

- 3 ladles water
- 3 Tbs soybean puree
- 1 ladle corn dough
- 1 tbsp. vegetable oil
- 1 tbsp. moringa juice

Ingredients

- 3 ladles water
- 4 tbsp. Bambara bean puree
- 3 tbsp. OFSP puree
- 1 ladle corn dough
- 1 tbsp. vegetable oil

Method

- Heat oil
- Boil 2 ladles of water.
- Add soybean puree to boiling water and stir until it thickens.
- Mix remaining water with corn dough to form paste.
- Add paste to mixture on fire and stir to form porridge.
- Add moringa juice to porridge and mix.
- Leave to simmer for 3 mins.

18. Corn/Bambara beans/Moringa

Ingredients

- 3 ladles water
- 4 tbsp. Bambara bean puree
- 1 tbsp. moringa juice
- 1 ladle corn dough
- 1 tbsp. vegetable oil

Method

- Heat oil
- Boil 2 ladles of water.
- Mix remaining water with corn dough to form paste, add salt.
- Add paste to mixture on fire and stir to form porridge.
- Add Bambara beans to porridge and mix.
- Add moringa juice to porridge and mix.
- Leave to simmer for 3 mins.

19. Corn /Peanut paste/Moringa

Ingredients

- 3 tbsp. of peanut paste
- 1 ladle corn dough
- 3 ladles water
- 1 tsp moringa powder

Method

- Pour 2 ladles of water in pot and boil.
- Add peanut paste and stir.
- Mix corn dough with water until smooth paste.
- Add to peanut mixture on fire.
- Stir till it thickens to form porridge.
- Stir in moringa powder and leave to simmer for 3 mins.

20. Millet/OFSP

Ingredients

- 2 ladles millet water (strained from millet dough after leaving it to settle for some time)
- 1 ladle millet dough
- 3 tbsp. of OFSP puree
- 1 tbsp. vegetable oil

Method

- Heat oil
- Add OFSP puree and millet water and boil for 3 mins.
- Add millet dough and stir over low heat to form porridge.
- Leave it to simmer for 2 mins.

21. Millet/soybean

Ingredients

- 2 ladles millet water
- I ladle soybean puree
- 1 ladle millet dough
- 1 tbsp. vegetable oil

Method

- Heat oil
- Add 2 ladles of millet water to oil and leave to boil.
- Add soybean puree to boiling water and stir until it thickens.
- Add millet dough and stir to form porridge.
- Leave to simmer for 2 mins.

22. Millet/Pawpaw

Ingredients

- 2 ladles millet water
- 1 ladle millet dough
- 3 tbsp. pawpaw puree
- 1 tbsp. vegetable oil

Method

- Heat oil
- Add pawpaw and millet water and boil for 3-5 mins
- Add millet dough and stir over low heat to form porridge
- Leave it to simmer for 2 mins

23. Millet/Bambara beans

Ingredients

- 2 ladles millet water
- 3 tbsp. Bambara bean puree
- 1 ladle millet dough
- 1 tbsp. vegetable oil

Method

- Heat oil
- Add millet water, Bambara beans and boil.
- Add millet dough and stir to form porridge.
- Leave to simmer for 2 mins.

24. Millet/Moringa

Ingredients

- 2 ladles millet water
- 1 ladle millet dough
- 1 tbsp. moringa juice
- 1 tbsp. vegetable oil

Method

- Heat oil
- Add pawpaw and millet water and boil for 3-5 mins
- Add millet dough and stir over low heat to form porridge
- Leave it to simmer for 2 mins

25. Millet/Peanut paste

Ingredients

- 3 tbsp. of peanut paste
- 3 ladles millet water (strained from millet dough after leaving it to settle for some time)

- 1 ladle millet dough

Method

- Pour millet water in pot and boil.
- Add peanut paste and stir.
- Add millet to peanut mixture on fire.
- Stir till it thickens to form porridge.
- Leave to simmer for 2 mins.

26. Millet/Cowpea

Ingredients

- 2 ladles millet water
- 1 ladle millet dough
- 3 tbsp. cowpea puree
- 1 tbsp. vegetable oil

Method

- Heat oil
- Add millet water, cowpea puree and boil.
- Add millet dough and stir to form porridge.
- Leave to simmer for 3 mins

27. Millet /Pawpaw/Moringa

Ingredients

- 1 ladle millet dough
- 7 tbsp. pawpaw puree
- 3 ladles of millet water
- A pinch of salt
- 1 tbsp. vegetable oil
- A pinch of moringa powder or 1 tbsp. moringa juice

Method

- Heat oil
- Add 2 ladles of millet water and boil.
- Add pawpaw and stir.
- Add salt to millet and mix with remaining water until smooth paste.
- Add to pawpaw on fire.
- Stir till it thickens to form porridge.
- Add moringa.
- Leave to simmer for 3 mins.

28. Millet/Pawpaw/OFSP

Ingredients

- 1 ladle millet dough
- 4 tbsp. OFSP puree
- 3 tbsp. pawpaw puree
- 3 ladles of millet water
- A pinch of salt
- oil

Method

- Heat oil
- Add 2 ladles of millet water and boil.
- Add OFSP and pawpaw purees and stir.
- Add salt to millet dough and mix with remaining water until smooth paste.
- Add to mixture on fire.
- Stir till it thickens to form porridge.
- Leave to simmer for 3 mins.

29. Millet/Pawpaw/Cowpea

Ingredients

Ingredients

- 1 ladle millet dough
- 3 tbsp. pawpaw puree
- 2 tbsp. cowpea puree
- 3 ladles of millet water
- A pinch of salt.
- 1 tbsp. vegetable oil

Method

- Heat oil
- Add 2 ladles of millet water and boil.
- Add pawpaw and cowpea purees and stir.
- Add salt to corn dough and mix with remaining water until smooth paste.
- Add to mixture on fire.
- Stir till it thickens to form porridge.
- Leave to simmer for 3 mins.

30. Millet/Pawpaw/Bambara beans

Ingredients

- 3 ladles of millet water
- 4 tbsp. Bambara beans puree
- 3 tbsp. pawpaw puree
- 1 ladle millet dough
- 1 tbsp. vegetable oil

Method

- Heat oil
- Boil 2 ladles of millet water.
- Add pawpaw puree to boiling water.
- Mix remaining water with millet dough to form paste, add salt.
- Add paste to mixture on fire and stir to form porridge.
- Add Bambara beans to porridge and mix.
- Leave to simmer for 2 mins.

31. Millet/ Pawpaw/Soy beans

Ingredients

- 1 ladle of millet dough
- 4 tbsp. pawpaw puree
- 2 tbsp. soy bean puree
- 3 ladles of millet water
- A pinch of salt.
- 1 tbsp. vegetable oil

Method

- Heat oil
- Add 2 ladles of millet water and boil.
- Add pawpaw and soy bean purees and stir.
- Add salt to millet dough and mix with remaining water until smooth paste.
- Add to mixture on fire.
- Stir till it thickens to form porridge.
- Leave to simmer for 3 mins.

32. Millet/OFSP/Moringa

Ingredients

- 1 ladle millet dough
- 4 tbsp. OFSP puree
- 3 ladles of millet water
- 1 tbs moringa juice
- A pinch of salt.
- 1 tbsp. vegetable oil

Method

- Heat oil
- Add 2 ladles of millet water and boil.
- Add OFSP purees and stir.
- Add salt to millet dough and mix with remaining water until smooth paste.
- Add to mixture on fire.
- Stir till it thickens to form porridge.
- Add moringa juice to porridge and mix.
- Leave to simmer for 3 mins.

33. Millet/OFSP/Cowpea

Ingredients

- 1 ladle millet dough
- 4 tbsp. OFSP puree
- 2 tbsp. cowpea puree
- 3 ladles of millet water
- A pinch of salt.
- 1 tbsp. vegetable oil

Method

- Heat oil
- Add 2 ladles of millet water and boil.
- Add OFSP and cowpea purees and stir.
- Add salt to corn dough and mix with remaining water until smooth paste.
- Add to mixture on fire.
- Stir till it thickens to form porridge.
- Leave to simmer for 3 mins.

34. Millet/OFSP/Bambara beans

Ingredients

- 1 ladle corn dough
- 4 tbsp. OFSP puree
- 2 tbsp. Bambara beans puree
- 3 ladles of millet water
- A pinch of salt.
- 1 tbsp. vegetable oil

Method

- Heat oil
- Add 2 ladles of water and boil.
- Add OFSP and Bambara beans purees and stir.
- Add salt to millet dough and mix with remaining water until smooth paste.
- Add to mixture on fire.
- Stir till it thickens to form porridge.
- Leave to simmer for 3 mins.

35. Millet/OFSP/Soy beans

Ingredients

- 1 ladle millet dough
- 4 tbsp. OFSP puree
- 2 tbsp. soy bean puree
- 3 ladles of millet water
- A pinch of salt.
- 1 tbsp. vegetable oil

Method

- Heat oil
- Add 2 ladles of water and boil.
- Add OFSP and soy bean purees and stir.
- Add salt to millet dough and mix with remaining water until smooth paste.
- Add to mixture on fire.
- Stir till it thickens to form porridge.
- Leave to simmer for 3 mins.

36. Millet/Soybeans/Moringa

Ingredients

- 3 ladles of millet water
- 3 Tbs soybean puree
- 1 ladle millet dough
- 1 tbsp. vegetable oil
- 1 tbsp. moringa juice

Ingredients

- 3 ladles water
- 4 tbsp. Bambara bean puree
- 3 tbsp. OFSP puree
- 1 ladle millet dough
- 1 tbsp. vegetable oil

Method

- Heat oil
- Boil 2 ladles of millet water.
- Add soybean puree to boiling water and stir until it thickens.
- Mix remaining water with millet dough to form paste.
- Add paste to mixture on fire and stir to form porridge.
- Add moringa juice to porridge and mix.
- Leave to simmer for 3 mins.

37. Millet/Bambara beans/Moringa

Ingredients

- 3 ladles of millet water
- 4 tbsp. Bambara bean puree
- 1 tbsp. moringa juice
- 1 ladle millet dough
- 1 tbsp. vegetable oil

Method

- Heat oil
- Boil 2 ladles of millet water.
- Mix remaining water with millet dough to form paste, add salt.
- Add paste to mixture on fire and stir to form porridge.
- Add Bambara beans to porridge and mix.
- Add moringa juice to porridge and mix.
- Leave to simmer for 3 mins.

38. Millet /Peanut paste/Moringa

Ingredients

- 3 tbsp. of peanut paste
- 1 ladle millet dough
- 3 ladles of millet water
- 1 tsp moringa powder

Method

- Pour 2 ladles of millet water in pot and boil.
- Add peanut paste and stir.
- Mix millet dough with water until smooth paste.
- Add to peanut mixture on fire.
- Stir till it thickens to form porridge.
- Stir in moringa powder and leave to simmer for 3 mins.
